# Appropriate solid waste management system in Quelimane (Mozambique): study and design of a small-scale center for plastic sorting with wastewater treatment

**DOI:** 10.1007/s42768-022-00091-6

**Published:** 2022-02-21

**Authors:** Francesca Villa, Giovanni Vinti, Mentore Vaccari

**Affiliations:** 1 Department of Civil and Environmental Engineering (DICA), Politecnico di Milano, Via Golgi 39, 20133 Milan, Italy; 2 Laboratory Centre on Appropriate Technologies for Environmental Management in Resource-Limited Countries (CeTAmb LAB), University of Brescia, Via Branze, Brescia, Italy

**Keywords:** Low-income countries, Wastewater treatment, Plastic recycling, Informal sector, Waste collection

## Abstract

Appropriate solid waste management (SWM) strategies are necessary to avoid severe environmental and sanitary impacts, especially in low-income countries. Such strategies are most likely to succeed whether implementing actors are supported by scientific research. In this paper, the results of a collaboration between local authorities and researchers are presented and discussed that are the assessment of waste generation in the city of Quelimane (Mozambique), integrating existing and field-collected data and the design of a small-scale center for plastic sorting to complement the SWM system of the city. The center is expected to receive about 0.3–0.4 t/day of plastic waste (5%–7% of the overall amount of plastic waste daily produced in Quelimane). As long-term sustainability represents a typical issue, simplicity of operation was a leading principle in the design of the center; moreover, the design included a treatment plant (WWTP) for generated wastewater, whose management is usually neglected in such interventions. Among others, natural wastewater treatment (constructed wetlands) has been chosen for its affordability. Noteworthy, the so-conceived WWTP appears as a novelty in the scientific literature associated with small-scale plastic sorting plants. The system is designed to treat an average flow of 6 m^3^/day and consisted of a septic tank followed by a subsurface flow constructed wetland. Overall, the COD (chemical oxygen demand) and TSS (total suspended solids) removal higher than 80% and 90% were estimated, respectively. Based on this work, both the center and the WWTP were successfully realized, which are waiting to become operational. In the authors’ opinion, the implemented procedure could become a reference for broader investigations and surveys.

**Supplementary Information:**

The online version contains supplementary material available at 10.1007/s42768-022-00091-6.

## Introduction

In many low- and middle-income countries (LMICs), waste management constitutes a big issue in all their stages that affecting the environment and public health [1]. Obstacles in establishing an effective and efficient solid waste management (SWM) system are mainly linked to social, financial, institutional, and organizational aspects [2, 3]. In this framework, Non-Governmental Organizations have started to implement strategies considering not only the stage of the collection but also reuse, recycling, and recovery alternatives, according to the waste hierarchy [4, 5]. The recovery of recyclable materials that operated by waste management authorities and  the informal sector can have at least two positive local impacts: first, it leads to a reduction in the amount of waste reaching the final disposal (often improper dumping, as in the case of Quelimane); then, it can constitute a source of income [6].

Among waste fractions, plastic poses many threats to health and the environment when not adequately managed, mainly in LMICs [7]. Indeed, in many LMICs data are merciless. For instance, Africa has the world’s highest rate of unsoundly disposed of plastic waste (on average, almost 90%) [8]. Furthermore, in many LMICs, less than 10% of the generated plastic wastes are recycled [9]. Unsurprisingly, plastic waste has been affecting the Oceans [10] with a significant contribution coming from LMICs [11].

In general, many approaches can be applied in plastic waste management [12]. Due to the high calorific value and mainly in industrialised countries, plastic waste is often used as a fuel. In many cases, the plastic waste  is used in waste incinerators along with other waste fractions [13]. However, possible adverse health outcomes must be kept in mind [14] even if risks are lower than uncontrolled waste combustion [7]. A promising alternative is represented by the pyrolysis of plastic waste, which has a lower carbon footprint compared to incineration [15]. In addition, pyrolysis has recently been studied at the laboratory scale by Veksha et al. [16] for marine plastic waste management. Furthermore, recent researches have highlighted how the conversion of plastics into valuable carbon products, such as carbon nanotubes, can serve as a sustainable way of waste recycling [17, 18]. However, the fact  that too-advanced approaches may not appear sustainably in LMICs should be highlighted [19]. With this in mind, some researchers have proposed appropriate solutions in low-income settlements, such as plastic-bonded sand paver blocks in Ghana [20] and Cameroon [21]. Plastic recycling can also constitute a job opportunity in both industrialised and developing countries, and even in rural areas of LMICs. In Asia, the case of Vietnam is emblematic: Salhofer et al. [6] analysed two rural settlements in which informal plastic waste (mainly polyethylene (PE) and polypropylene (PP)) recycling was carried out by local craftsmen. Such activity played an important role in contributing to rural socio-economic development.

When it comes to the introduction of the plastic waste recycling initiative, many aspects should be considered. First, it is essential to understand that the first step in reducing plastic waste dumping must aim at growing the waste collection which is very low in many LMICs [22]. Then, plastic recycling that may cause environmental and health threats ( as described in [6]) should be targeted.

For example, waste mismanagement and contaminated water are interlinked problems [23]. In many cases, wastewater or leachate is not treated, and is directly discharged to open channels, water receptors, or the environment [23–26]. WHO et al.  [27] estimated that globally about 750 million people, of whom over 90% lived in urban areas, had sewer connections that did not receive adequate treatment. LMICs are affected seriously by this issue. For instance, in Latin America and Caribbean, only about 30% of the wastewater is collected and  treated [28]. However, several LMICs such as Mozambique lack detailed information on wastewater treatment [27].

This manuscript focuses on a small-scale center for plastic sorting (CPS), which will be basically operated by a group of workers, and most of the operation will be manually. According to Vest [29], those characteristics can make such a kind of plant appropriate to a low-income context, together with its environmental friendliness. Consequently, the design of the center includes a wastewater treatment plant (WWTP) to tackle properly the existing risk of contamination [30]. Unfortunately, even the centers for manual plastic sorting exist in Mozambique and many parts of the world, information about their operation and management, especially concerning wastewater treatment, is lacking. Most information on the design of such a wastewater treatment plant is related to the industrial treatment of plastic, which represents a different process. To the best of our knowledge, only [31] discussed the characterization of effluents through a plastic recycling process in the scientific literature; however, in the case that a similar layout was in place, the presence of cleaning agents in the effluent made the comparison difficult.

Regarding the choice among different wastewater treatment technologies, many of them may be considered appropriately for developing countries [32]. Among them, natural wastewater treatment systems that characterized by using the soil and/or plants to sustain microbial populations treat wastewater in a relatively passive manner. In most cases, this kind system is not only less expensive and easier to maintain than classical WWT plants, but also less energy-demanding than mechanical treatment alternatives [33]. One of the most interesting natural treatments is represented by constructed wetlands (CW), through which the function of the natural wetland is emulated and improved, and the removing of contaminants depends on the synergistic effects of the substrate, microorganism, and plant in physics, chemistry, and biology [34]. It is important to note that despite CW representing a recent technology with a few decades of life [35], it has already extended diffusion in developing countries in which to treat domestic sewage and other types of polluted water such as industrial wastewater and landfill leachate [36]. Lamentably, we have not found any scientific publication concerning natural wastewater treatment systems in Mozambique.

## Context

### Solid waste management (SWM) in Mozambique: state-of-the-art

Mozambique, which went through a civil war and reached partial political stability only in 1994, is still one of the poor countries in the world and ranks 180 of 189 countries and territories in the the Human Development Index [37]. Since 1999, when Municipalities (*Autarquias*) were introduced, most public services were decentralized without the proper transfer of capacities or financial resources [38] which are  still lacking in the waste management sector until now.

The legal framework for SWM in Mozambique is defined by the National Strategy for Integrated SWM, that was prepared in 2012 by the Ministry for Coordination of Environmental Affairs [39] and the Regulation on Municipal SWM, and then issued by the Government in 2014 [40]. The framework identifies Municipal Councils and Districts as the main actors of the system. The main objectives for the National Strategy are: (1) to  strengthen of the SWM system (capacity building within public institutions, creation of management plans); (2) to  promote the reduction, reuse, recycling, and composting of waste and  improve the collection and the creation of sanitary landfills; (3) to  establish partnership among public actors, private actors, and the civil society; (4) to implement a monitoring system [39, 41].

According to [39] and [40], Municipalities have the responsibility for waste management and are in charge of the preparation and implementation of the local SWM plan including standards and guidelines for the separate collection.

Within this framework, several problems exist: SWM Plans are absent in most cities of Mozambique, data are insufficient to design a proper system, and the economic coverage is not guaranteed by municipal fees [4]. Waste collection mainly focused on urban areas with about 40%–65% population according to [39, 41]. With regards to waste treatment, some initiatives for composting and recycling are already in place, particularly in the capital city Maputo [42], and a secondary raw materials market is available in the country. Anyway, these initiatives are not within a coordinated framework, and  are mostly run by civil society organizations (CSOs) or the private sector [39, 41]. Dumpsites (*lixeiras*) which are characterized with open burning and no containment in place are the primary option for final disposal. Even a formal inventory for dumpsites does not exist, dumpsites can be found in the proximity of almost every city or settlement [39, 41]. As required by the National Strategy [39], almost each city has identified a location for a sanitary landfill, and has performed site evaluations and Environmental Impact Assessments (EIAs) [41]. The informal sector including both civil society organizations and waste pickers (*catadores*) is available in the country that performs collecting recyclables such as plastic, glass, and metals [39, 43].

### The case study: Quelimane

Quelimane is the capital city of Zambesia, a region in the north of Mozambique. The population is about 350,000 [44]. The city locates at a flat area near the sea with the  southern boundary represented by the river Rio Dos Bons Sinais.

**Fig. 1:**
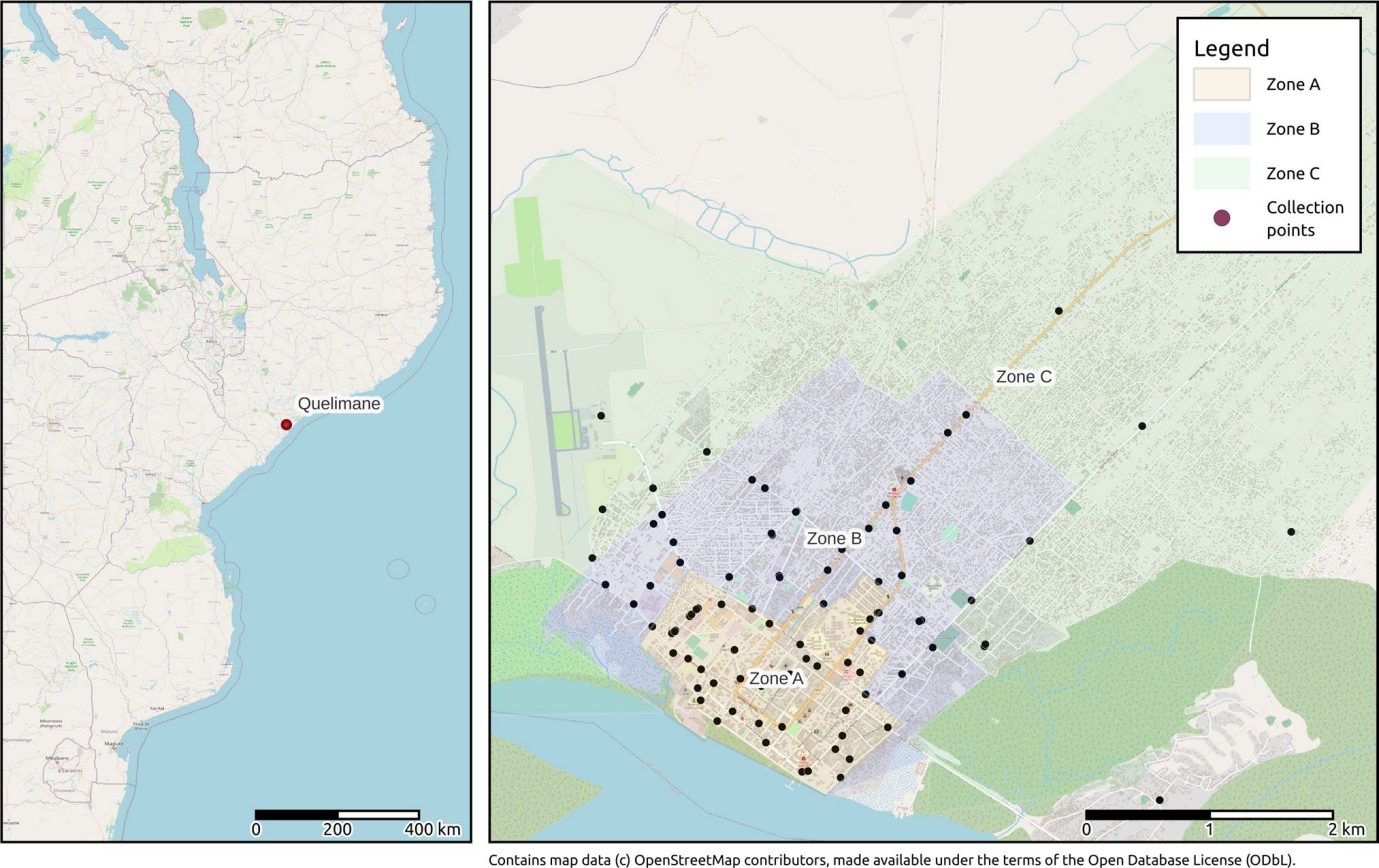
The city of Quelimane: geographical position, tentative identification of Zones (A—Cidade cemento, B—urban suburbs, C— rural area) and location of collection points (Basemap: OpenStreetMap [47])

The first SWM Plan of Quelimane was done in 2004, while the municipal company EMUSA was established in 2005. The present SWM Plan [45] provides a picture of the functioning system function. In this study, the city is divided into three areas by the local NGO Amor [46] based on its survey of the  household waste generation: the center, or “cidade cimento” (Zone A, 10,495), the urban suburbs (Zone B, 174,921) and the rural area (Zone C, 164,426). The waste management operated by EMUSA does not cover all urban suburbs and rural areas (Fig. 1), while  the service in the city center lacks efficiency and efficacy. This fact is mainly due to financial and organizational burdens. Other treatments are missing even if informal waste pickers are available to collect recyclables from street containers or in the dumpsite. The collected waste is finally disposed at an improper dumpsite located in a wetland. An area for the establishment of a controlled landfill has been identified, but it is not clear if the project will have any continuation in the short-middle term.

### The project “Quelimane Limpa”

The project “Quelimane Limpa” (which means “Quelimane Clean” in Portuguese) was realized by the Municipality of Quelimane that was supported by an Italian Non Governmental Organization (NGO) and in partnership with local associations. The project started in December 2016 and lasted until August 2019.

The objective of the project (coherent with [39]) was to improve the hygienic and environmental condition of the city of Quelimane by enhancing the cooperation among local authorities, civil society, and private companies in the sector of waste management. Several actions have been applied to achieve this objective. The first set of actions had its specific objective to  empower the municipal company EMUSA through training its managers, employees, and workers with the provision of equipment, technical support in re-organizing the SWM system of Quelimane. The second set of actions dealt with raising of citizens’ awareness through schools and civil society organizations (CSOs). The third set of actions was expected to create economic activities in the sector of waste recycling to promote employment and entrepreneurship within the waste management sector. This strategy was based on the construction of two small-scale plants, a composting center (CC) and the CPS presented in this study, and the creation of small groups of workers that defined as “micro-enterprises” charged with the responsibility of recyclables collection and management of the two centers. If the initiative is successfully realized, it represents an opportunity for social inclusion that  provides some tools or at least a point of reference to the active waste pickers. It is worthy to note that Ref. [39] mentions *catadores* as the main partner for the establishment of the separate collection even with the responsibility of organizing themselves in associations.

The construction of two centers was completed in February 2019 (CC) and in July 2019 (CPS), respectively. Meanwhile, three micro-enterprises of five people were created. All the workers of the micro-enterprises underwent specific training on composting methods and plastic identification. They started operations in 2018 including collecting and transforming organic waste into compost, and collecting and selling separately plastic waste.

## Materials and methods

For the first step, an assessment of municipal solid waste (MSW) generated in Quelimane together with its composition and distribution within the city has been done. The assessment results represent the most recent information about MSW for the city and the only attempt to obtain a characterization of non-domestic waste as follows. The CC and CPS has been consequently designed based on these results. These activities were the authors’ main contribution to the project “Quelimane Limpa” as well as the constant remote and field support on technical and organizational issues.

### Generation and distribution of MSW in Quelimane

When dealing with low-income countries, detailed data collection is the main issue. Existing data provided by local studies have been used for the estimation of MSW generation in Quelimane.

#### Generation of MSW waste

The first data source is the MSW Management Plan of Quelimane [45]. The waste daily generation is presented in the Plan regarding on the different waste sources (domestic, markets, commercial and institutional, tourism, and street sweeping). Domestic waste generation is estimated at 107.4 tons/day ( with a population of 231,017), including waste released from households and the maintenance of Public green.  The forecasting annual increase rates are provided for the domestic waste in the following table. Note that the street sweeping wastewas assumed by [45] as constant, whereas the authors decided an increase of 2.6% which equals to the annual average growth of population (Table 1).

**Table 1:** Production of MSW (municipal solid waste) in Quelimane [45]

Source	Daily production in 2013		Annual increase
Domestic waste	(ton/day)	(% on the total)	%
City center	7.2	6%	1
Suburbs (high density)	48.3	42%	4
Suburbs (middle density)	45.1	39%	4
Rural areas	6.8	6%	− 1
Markets	1.1	1%	5
Commercial and institutional	5.8	5%	3
Tourism	1.2	1%	5
Street sweeping	0.5	0.4%	2.6^a^
Total	116	100%	

^a^Decided by the authors

#### Generation of household waste

During the period of this research, the local NGO Amor published a study on the composition of MSW in Quelimane [46] collaborated with the municipal company EMUSA. The NGO study analysed 91 households samples with interviews the socio-economic characterizations and providing with two buckets for the wet and dry waste, respectively. The waste produced by each household was weighted on a daily base.  Based on the composition analysis, it showed that the daily generation per capita of household waste (HH) was 0.39 kg/inhab/day for Zone A , 0.31 kg/inhab/day for Zone B, and 0.30 kg/inhab/day Zone C. The average number for Quelimane was 0.31 kg/inhab/day, and the daily generation of HH was at 107.6 tons/day.

#### Final disposal

The final disposed waste amount can be calculated based on  daily trip number to the dumpsite, which has been estimated at 20 trips per day by EMUSA [46]. Each trip load is about 3 tons since the skip trucks used for the final disposal can load a 6 m^3^ container. The not collected waste amount will be calculated by MSW generation.

### Composition of MSW in Quelimane

Data for Quelimane come from the research done by Amor [46], in which the household waste generation was daily sampling of one week for 91 households located in all the city  with 1301 kg sample in total. The data are shown in Table 2, including the separation between Food waste and dry waste, divided into remaining food waste (4%), paper/cardboard (6%), plastic (20%), glass (43%), metal (13%), tissues (6%), others (0.14%).

**Table 2:** Composition of Household waste in Quelimane [46]

Zone	Income	Number of samples	Dry waste (kg/inhab/day)	Food waste (kg/inhab/day)	Total (kg/inhab/day)
A	Middle-high	21	0.06%	0.33	0.39
B	Middle-low	47	0.07%	0.24	0.31
C	Low	23	0.07%	0.23	0.3

The following information has been crossed with  field surveys (FS) done by the implementing NGO with the local team and authors’ support:

- Commercial (FS_L): detailed characterization of dry waste collected from 13 shops of the city center, one week, April 2019 (49 kg  totally).
- Markets (FS_Mark): 3 samples from street containers of 3 tons located at 3 different markets (Chabeco, Central, Aquima), May 2017 (about 1 ton per sample, 3258 kg totally).
- Mixed (FS_Mix): 3 samples (about 1 ton each) from street containers of 3 tons located at commercial/residential areas, May 2017 (about 1 ton per sample, 2853 kg totally).
- Mixed (FS_Ce): detailed analysis of the whole contents of 6 street bins (0.06 m^3^) located at the city center, April 2018 (101 kg totally).
- Final disposal (FS_At): at the entrance of the dumpsite, 2 samples per day with 5 days sampling, May 2017 (about 100 kg per sample, 1232 kg totally).

Different methods have been used due to typical constraints of development cooperation projects. The reliability of the results will be discussed in the following section together with their presentations.

### Design of the CPS

The CPS is supposed to receive only plastic waste coming from the separate collection. The separate collection can be organized in several ways that involving the micro-enterprises and the informal sector. Since the CPS will be operated by a micro-enterprise created within the project whose workers have no technical skills and a low level formation, most operations have been planned to be manually or based on a simple machinery. The center has been designed according to a classical layout [48, 49], which includes the acceptance and sorting area, the primary storage area for loose material, the washing and drying area, the shredding area, and the secondary storage area for the treated material.

### Design of the wastewater treatment plant

The wastewater treatment plant of Quelimane has been designed to treat the wastewater that produced during the washing of plastic in the CPS.

The following three main types of constructed wetlands are taken into consideration [33]:

- The free water surface (FWS) wetland, which is similar to a natural marsh with the water surface exposed to the atmosphere;
- The subsurface flow (SSF) wetland, in which a permeable medium is used, and the water flow is horizontal while the water level is maintained below the top of the bed;
- The vertical flow (VF) wetlands, in which the distribution system is on the surface, and the distributed flow moves vertically through a permeable media.

The SSF wetland was selected for the wastewater treatment, considering the advantages that characterized CW as previously described. It is possible to design the SSF plant using detailed models and the related algorithms when enough and specific data are available [33, 50], otherwise, the  simplified and conservative models should be applied.

In any case, a CW is typically preceded by a preliminary treatment as an Imhoff tank or a septic tank to remove sedimentable solids so as to avoid clogging the constructed wetland downstream [32, 33]. Further preliminary treatments to remove oil and grease may be necessary as well.

For the present case study, very few literature data are available. Among them, a Brazilian study [31] considered the impacts of plastic waste treatment from an MSW separating and composting plant. The authors analysed the effluent from the pre-washing and rinsing steps and found that the characteristics of liquid effluents were equivalent to those in untreated domestic wastewater classified in a level between medium and strong.

 Due to lacking enough specific information, the CW has been decided to follow the conservative simplified design described by [51], in which a hydraulic detention time (HDT) of at least 4 days should be taken into consideration to respect the effluent limits as a reference. After a comparison with the Italian legislation [52] (based on European directives), threshold values of COD (chemical oxygen demand) and TSS (total suspended solids) from the Mozambican legislation [53] have been assumed. Usually, the depth of the bed has ranged from 0.3 m to 0.9 m [33]. It is possible to use Eq. (1) to find the volume (*V*):

1 $$V = Q \times HDT,$$

where: *V* = volume of the bed, *Q* = average flow through the wetland, *T* = hydraulic detention time.

To design the septic tank, Bonomo suggested to consider an HDT of 12 h with adding a further volume to allow the accumulation of sludge between two emptying which assuming a sludge production rate of 100 L/inhab/year [54].

The average flow (*Q*) will be defined considering the amount of material that will be treated within the CPS.

## Results and discussion

### Generation and distribution of MSW in Quelimane

The MSW daily production has been estimated at 133.2 t/day for the year 2017 including the following categories of waste: domestic, markets, commercial and institutional, tourism, and street sweeping. The calculated amount of domestic waste (123.3 t/day) was split by household waste at 107.6 t/day given by Amor [46] and Public green at 15.7 t/day. Concerning the final disposal, the collected waste released to the dumpsite is about  60 t/day, while the not-collected waste is about 73 t/day. This means the collection rate is about 45%, which constitutes a slight increase from 34% that provided by the PGIRSU [45]. Results are presented in Table 3.

**Table 3:** Production and final disposal of MSW on a daily basis, total amount (2017)

Production of waste	Amount (t/day)	Source of data	Composition
Household waste	107.6	Amor [48]	Amor [48]
Public green	15.7	Amor [48], PMGIRSU (2013)	Organic (wood)
Markets	1.3	PMGIRSU [46] (forecast 2017)	FS_Mark
Commercial and institutional	6.5	PMGIRSU [46] (forecast 2017)	FS_L, FS_Mix
Tourism	1.5	PMGIRSU [46] (forecast 2017)	FS_L, FS_Mix
Street sweeping	0.6	PMGIRSU [46] (forecast 2017)	FS_Ce
Total production	133.2		(calculated)

Final disposal	Amount (t/day)	Source of data	Composition
Dumpsite	60.0	Number of trucks (3 tons capacity)	FS_At
Unknown destination	73.2	Total production—dumpsite	(Calculated)

### Composition of MSW in Quelimane

The amount of each fraction has been calculated based on the existing data on household waste composition [46] and field surveys. Data from field surveys have been aggregated regarding fractions used by Amor: organic (food waste), organic (wood), paper/cardboard, plastic, glass, tissue, metal, and the others. For household waste, the composition proposed by Amor was used. Commercial and institutional waste was combined with Tourism in a new category (Mixed), for which the composition was calculated from both commercial waste (FS_L) and street containers (FS_Mix) field survey. Since the workers are used to dispose the collected waste in bins, the composition FS_Ce has been used for Street sweeping. For other categories, the composition was calculated based on field surveyswhich has been shown in Table 2. The final results are presented in Table 4.

**Table 4:** Production and final disposal of MSW on a daily basis, composition (2017)

Item	Production (t/day)						Final disposal (t/day)	
	Household	Mixed	Public green	Markets	Street sweeping	Total	Dumpsite	Unknown destination
Organic (food waste)	84.23	5.02	NA	1.12	0.47	90.85	44.28	46.56
Organic (wood)	NA	0.38	15.71	0.04	0.02	16.15	3.20	12.95
Paper/cardboard	1.46	0.74	NA	0.04	0.04	2.29	2.61	− 0.32
Plastic	4.86	0.90	NA	0.04	0.02	5.82	3.78	2.04
Glass	10.46	0.24	NA	0.02	0.01	10.72	1.76	8.96
Tissue	NA	0.33	NA	0.03	0.01	0.36	0.51	− 0.14
Metal	3.16	0.23	NA	0.01	0.00	3.40	1.12	2.27
Other	3.40	0.16	NA	0.02	0.02	3.61	2.74	0.86
Total	107.58	8.00	15.71	1.30	0.60	133.19	60.00	73.19

*NA* not available data

The organic fraction that serves as  the target material for the organic center is abundant: food and green is estimated at 68% of daily production, whether the wood amount is about 12%. Other fractions have been estimated as follows: the amount of paper and cardboard is 1.7%, the amount of metals is 2.6%, while the amount of glass is 8%. Finally, the calculated amount of plastic is less than 5% (5.82 t/day).

In terms of comparison, the average values for low-income and low-middle income countries [22] and specific values of  Kampala (Uganda) [55] have been taken as the reference which are shown in Table 5. The organic fraction of MSW always takes  more than 50%, while other components, i.e. paper and cardboard (lower than compared values), and glass present significant differences. Nonetheless, specific living conditions and consumption patterns can influence local values as clearly shown by MSW composition in Kampala.

**Table 5:** Comparison of MSW composition in similar contexts: Quelimane (Mozambique), Kampala (Uganda), and average values for low-income and low-middle income countries (NA = not available data)

Waste component	Quelimane (Mozambique)	Kampala (Uganda) [55]	Average values [22]	
			Low-income countries	Low-middle income countries
Food and green [%]	68.2	83.0	56.0	53.0
Paper and cardboard [%]	1.7	5.0	7.0	12.5
Metal [%]	2.6	1.0	2.0	2.0
Plastic [%]	4.4	8.0	6.4	11.0
Glass [%]	8.1	1.0	1.0	3.0
Rubber and leather [%]	NA	NA	<1.0	<1.0
Wood [%]	12.1	NA	<1.0	1.0
Other [%]	2.9	2.0%	27.0	17.0

#### Plastic waste: composition and local market

In some field surveys, the much detailed information concerning plastic types has been reached. The information shows that the rate of  the plastic fraction for polyethylene terephthalate (PET) is 10%, while  55% for HDPE (high-density polyethylene), 7% for polystyrene (PS), 3% for low-density polyethylene (LDPE). The results are presented in Table 6. They are not entirely reliable due to the typical uncertainty in plastic identification that carried out by a non-professional team.

**Table 6:** Plastic types in the samples

Item	FS_3t (mix+mark)	FS_Ce	FS_L	FS_At	Average	Marketable or not
Plastic (%) in the sample	27.48%	14.15%	13.82%	6.30%		
PET						
Bottles	6.07%	8.60%	14.41%	12.37%	10.36%	No
HDPE						
Hard plastic	36.27%	11.24%	4.53%	17.40%	17.36%	Yes
Bottle caps	11.57%	0.83%	0.45%	12.50%	6.34%	Yes
Bags	11.02%	27.27%	14.93%	17.14%	17.59%	Yes
Film	21.23%	11.57%	0.37%	19.72%	13.22%	Yes
PP						
Hard plastic	0.00%	0.50%	0.89%	0.00%	0.35%	Yes
Film	0.00%	8.10%	22.07%	0.00%	7.54%	No
PS						
Packaging	4.33%	2.81%	13.89%	7.86%	7.22%	No
LDPE						
Film	0.00%	4.13%	7.50%	0.00%	2.91%	Yes
Others						
Food packaging	0.00%	7.60%	0.22%	0.00%	1.96%	No
Film PC	0.00%	5.12%	0.00%	0.00%	1.28%	No
Composite packaging	0.00%	6.28%	12.18%	0.00%	4.62%	No
Film others	9.49%	2.48%	8.54%	13.02%	8.38%	No
Hard plastic	0.00%	3.47%	0.00%	0.00%	0.87%	No
Total	100.00%	100.00%	100.00%	100.00%	100.00%	57.77%

*PET* polyethylene terephthalate,* HDPE* high-density polyethylene,* PP* polypropylene,* PS* polystyrene,* LDPE* low-density polyethylene,* PC* Polycarbonate

A rough average which can be assumed as a qualitative indication has been calculated for each type of plastic. A detailed model should be elaborated considering the variation among several stages. For example, the plastic amount in the waste releasing the dumpsite is low, which is probably due to the action of street waste pickers.

In the project, an assessment considered the accessibility on the plastic market has been done. The local market should be preferred even considering the bad condition of roads and the long distances characterizing the country. The result showed  that a local plastic market currently exists only in hard HDPE, PP, HDPE film, and LDPE film, which accounts for 58% out of 5.82 t/day of plastic waste daily produced in Quelimane. The local market includes  one manufacture in Quelimane which produces plastic products and uses both virgin and recycled plastic (currently purchases from South Africa and Portugal), and other industries in Beira which are about 500 km from Quelimane. All these actors are interested in buying recycled plastic and prefer clean and shredded plastic waste.

The plastic waste share that can be sold on the local market is shown in Table 6 as “Marketable” (fit for sale). This information has been used to assess the economic sustainability of the project since the marketable share of the plastic waste overall amount will contribute to the CPS earnings.

Finally, a hypothesis on the presence of plastic in Quelimane was applied concerning the solid waste amount produced based on both households and non-residential sources. Results are shown in Table 7.

**Table 7:** Presence of plastic with reference to different zones in Quelimane

Sources	Total amount of plastic waste (t/day)	Amount of plastic waste with an accessible market (t/day)
Households		
Zone A	0.11	0.07
Zone B	2.45	1.41
Zone C	2.30	1.33
Non-residential sources	0.96	0.55
Total	5.82	3.3

### Design of the CPS

The previously presented data have been used as a basis for the small-scale center design of plastic sorting. The center is supposed to host the plastic waste treatments for primary storage, manual separation, shredding, washing, drying, and secondary storage.

According to the project, the center would receive plastic waste from 6 micro-enterprises and 10–13 workers will be fully assigned to the plastic waste collection and the management. Based on the economic calculations of the minimum wage in Mozambique, each worker should collect about 30 kg plastic each day, while 17 kg of that are considered as marketable. Consequently, the center has been designed to receive 0.3–0.4 t/day of plastic waste. This capacity will be enough for the plastic collected in Zone A where about 0.1 t/day of plastic waste is produced (as shown in Table 7). Besides, plastic should be collected from nearer suburbs to reach the required amount. After the manual selection that targets the plastic types previously listed, the marketable plastic amount subjected to subsequent treatments and storage ranges from 0.17 t/day to 0.23 t/day. The remaining plastic would be discarded.

**Fig. 2:**
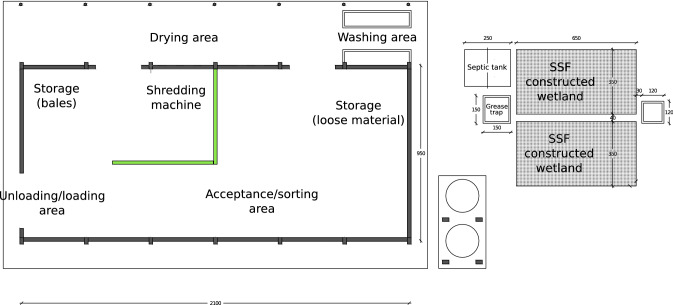
Layout of the center for plastic sorting. *SSF* Subsurface flow

The layout of the center is shown in Fig. 2. The main building is composed of a warehouse (190 m^2^) and an open area. The functional areas in the warehouse are divided according to mobile walls to adapt to the operational needs. Regarding primary storage, the density of the loose material is supposed to be 0.01–0.02 t/m^3^ depending on the quantity of each fraction [56]. In this case the primary storage will occupy 70 m^2^ to 100 m^2^ considering 7 days of storage. The supposed density of secondary storage of the treated plastic rangs from 0.2 t/m^3^ to 0.3 t/m^3^ whether shredded or baled  [57, 58]. The secondary storage will occupy 30 m^2^ to 50 m^2^ considering 30 days of storage. The remaining space will perform the other functions, such as the unload/load of the vehicle, the acceptance/sorting, and the operation of the shredding machine. The open area (a covered courtyard) will host the manual washing and drying process.

 Two steps for manual washing of plastic material has been designed based on two washing tanks. Each tank will be split vertically into two parts with a grid which will separate the plastic from the dirty sediment. The hard plastic will be shredded before the washing stage, while the film will be washed directly after the selection stage. Each batch has been assumed to be about 15 kg which is lifted by the operator with a minor effort. Thus, the minimum volume of one steady batch is about 0.75 m^3^ (assuming a density of 0.02 t/m^3^). Consequently, the two tanks have been designed to contain 1.5 m^3^ of water, which will be completely replaced twice per day. Accordingly, the average flow is assumed to be 6 m^3^/day.

### Design of the wastewater treatment plant

The washing tank connects to a septic tank, a grease trap, and an Subsurface Flow wetland (SSF) constructed wetland at the end (see Fig. 2). As previously discussed (see “Design of the CPS”), the average flow is  6 m^3^/day. As a consequence, the volume of the sedimentation zone of the septic tank is calculated as follow:

2 $$V_{{{\text{sed}}}} = \frac{Q \times HDT}{{24}} = \frac{{6\,{\text{m}}^{3} /{\text{day}} \times 12\,{\text{h}}}}{{24\,{\text{h}}}} = 3\,{\text{m}}^{3} ,$$

where: *V*_sed_ = volume of sedimentation zone of the septic tank. *Q* = average daily flow through the septic tank.

It can be noted that Eq. (2) is conceptually the same with  Eq. (1).

Furthermore, the amount 230 kg/day has been considered as the peak daily rate of plastic treated by the sorting plant. For the pollutant load in terms of COD expressed as g/kg of plastic, the value presented by [31] is incorporated. The most precautionary value is the sum of pre-washing and washing of HDPE and PP, i.e. 16.8 g/kg. As a consequence, the daily COD production is  calculated using a simple mass balance:

3 $${\text{COD}}_{{\text{d}}} = {\text{COD}}_{{{\text{rate}}}} \times M = 16.8\,{\text{g}}/{\text{kg}} \times 230\,{\text{kg}}/{\text{day}} = 3864\,{\text{g}}/{\text{day}},$$

where:

COD_d_ = daily COD produced with the wastewater; 

COD_rate_ = amount of COD per kg of plastic under pre-washing and washin;

*M* = upper daily rate of plastic washed in the sorting plant.

The corresponding COD concentration (COD_conc_) is 644 mg/L (i.e. COD_d_/*Q*).

Based on  the same procedure, the TSS influent concentration can be estimated:

4 $${\text{TSS}}_{{{\text{conc}}}} = \frac{{{\text{TSS}}_{{\text{d}}} }}{Q} = \frac{{{\text{TSS}}_{{{\text{rate}}}} \times M}}{Q} = \frac{{22\,{\text{g}}/{\text{kg}} \times 230\,{\text{kg}}/{\text{day}}}}{{6\,{\text{m}}^{3} /{\text{day}}}} = 843\,{\text{g}}/{\text{m}}^{3} = 843\,{\text{mg}}/{\text{L}}.$$

It has to be noted that conservative assumptions were made. Indeed, the peak  daily rate of plastic washed in the plant that we considered in the design allows us to guarantee the required effluent during the highest waste flow. As a result, effluent quality will be even better for most of the operational time. Assuming a COD value of 130 g per equivalent inhabitants (EI) per day [54], it is possible to find the equivalent inhabitants is related to the daily COD load as follows:

5 $${\text{EI}_{\text{COD}}} = \frac{{{\text{COD}}_{{\text{d}}} }}{{{\text{COD}}_{{{\text{EI}}}} }} = \frac{{3864\,{\text{g}}/{\text{day}}}}{{130\frac{{{\text{g}}/{\text{day}}}}{{{\text{EI}}}}}} = 30{\text{EI}},$$

where: EI_COD_ = equivalent inhabitants in function of the daily COD load. COD_EI_ = COD produced per equivalent inhabitant.

The volume of the sludge has been calculated considering 3 emptying every 2 years, resulting in 2 m^3^ as follows:

6 $$V_{{{\text{sludge}}}} = \frac{{{\text{EI}} \times S_{{{\text{rate}}}} }}{f} = \frac{{30{\text{EI}} \times 0.1\,{\text{m}}^{3} /{\text{year}}}}{{1.5\,{\text{times}}/{\text{year}}}} = 2\,{\text{m}}^{3} ,$$

where: *S*_rate_ = sludge production rate per inhabitant per year, which is assumed as 100 L/inhab/year according to Section “Design of the wastewater treatment plant”. *f* = frequency of  the emptying per year.

Thus, the total volume needed for the septic tank was returned by the sum of *V*_sed_ and *V*_sludge_ as 5 m^3^.

Regarding the SSF constructed wetland with an HDT of 5 days, the total volume of the bed is calculated as 30 m^3^ according to Eq. (1). Based on the values discussed in Section “Design of the wastewater treatment plant” [33], we fixed an bed intermediate depth as  0.7 m. Thus, the surface area of the wetland is calculated as:

7 $$A = V/d = 30\,{\text{m}}^{3} /0.7\,{\text{m}} = 43\,{\text{m}}^{2} ,$$

where: *A* = surface area of the wetland, *V* = volume of the bed, *d* = depth of the bed.

Afterward, the area  is increased a bit to 45.5 m^2^ due to safety reasons and the initial uncertainties. The peak precautionary value for pollutant load is taken from the only available research we found [31]. The slight additional increase we finally conceived will, in any case, guarantee an effective water treatment which will benefit both environmental and health. It is important to highlight the hydraulic profile of the plant such as gravity flow. As a consequence, the energetic requirement of the system during the operational phase is equal to zero. Energy would be needed only during sludge emptying of the septic tank and the maintenance operations. The emptying time of the septic tank depends on wastewater quality which needs even more than 5 years in some cases [59].

It is important to consider that septic tank units can achieve a removal of 50%–70% for TSS [60] and a removal of 50% for COD [61]. Furthermore, in CW designed in our system, a TSS removal of 70%–90% [51] and a COD removal higher than 50% and even up to  90% [62] can be expected. These values can be taken as reference limits based on the Mozambique legislation, i.e. COD_out_ = 80 mg/L, TSS_out_ = 30 mg/L. It has to be highlighted all the units will be monitored in the operational phase to compare the theoretical assumptions with the actual values of the plant following the little existed literature information.

Further considerations can be done in a long-term perspective. If the plant proves to be reliable and efficient, the plastic waste flow can be increased in the future. In this case, the WWTP should be expanded, too. For instance, if the plastic waste flow is doubled (i.e. 460 kg/day), it will lead to double the volume of the needed water cleaning the plastic waste (i.e. 12 m^3^/day). Using such new values in Eqs. (2), (5), (6), volumes required would double as well. In this case, new units in parallel with the existing ones could be conceived.

### Final achievements of the project

The CPS construction started at the beginning of 2018 and lasted about 6 months. It began to operate at least 1 year before the end of the project. This timeline was planned by the implementing NGO to consent further analysis on the functioning of the center and subsequent adjustments on its operational strategy. Unfortunately, the works were delayed for several reasons: firstly, both centers for composting and plastic sorting were supposed to be located at the same site of the sanitary landfill, and another location had to be selected which took too much time; secondly, the project encountered some organizational and administrative issues; thiredly, excavation works were delayed due to heavy rains which caused the raising of  the water table surface; finally, the situation was worsened by the massive meteorological events that  affected Mozambique in March and April 2019 (the cyclones Idai and Kenneth) [63] even Quelimane experienced light damages compared with other areas of the country. As a result, the center was  completed at the end of the project in July 2019. Furthermore, the purchased equipments (the press and the shredding machine) are still waiting to be delivered due to the impact of the COVID-19 pandemic. Annex 1 (Supplementary Materials) contains pictures of the center during several stages of its construction. Even if the center is still not fully operational, the plastic waste collection has been started based on  the work of 3 micro-enterprises which collected more than 4 tons of plastics in the last period of the project.  The plastic was  manually sorted and storaged at the center. Plastic was then sold to local industries manufacturing plastic products.

For the economic aspect of the projects, the construction works for both CPS and related WWTP cost about 26,000 €, while the purchase of the equipments (the press and the shredding machine) cost about 15,000 €. Thus, an overall capital cost is 41,000 €. According to the economic assessment, the market value for dirty plastic is about 74 € per ton, and  it can reach to  147 € per ton when plastic is shredded and washed. Considering a 10 years useful life for the center and the annual capacity about 110 tons per year of recycled plastic, it is possible to estimate a capital cost of 38 € per ton of recycled plastic. Being a development cooperation project, the capital cost has been covered by  grant instead of pay  back. The investment seems to be justified in any case besides the economic point. In fact, the construction of such a center not only implies an  organized, efficient, and environmentally sustainable system, but also enables  workers to perform their duties in a healthy way. In addition, the existence of a storage zone represents an opportunity to  increase negotiation power with plastic buyers. On the other hand, uncertainties are related to the effective use of the center, its integration with the SWM system of Quelimane, and the need for coverage of management costs which are usually absent in an informal organization.

## Conclusions

The purpose of this research was to provide scientific support to a Non-Governmental Organization that intended to realize a project in the sector of waste management in the city of Quelimane (Mozambique). At the first step, an assessment of the generation of MSW in Quelimane was done to get consistent data. The production of plastic waste in Quelimane has been estimated at 5.82 t/day (less than 5% of the overall production of solid waste), of which about 58% are with a market value. The second step was the design of a small-scale center for plastic sorting which aimed at receiving 5%–7% of the plastic and including a WWTP to mitigate the risk of environmental contamination. The requirements behind technical choices were the simplicity of operation and environmental sustainability. For this reason, most of the treatment stages were designed to be manually. Besides, the technology of the wastewater treatment plant (constructed wetlands) was chosen due to  its easiness of operation and affordability. The construction of the two infrastructures has been completed in July 2019 despite many organizational, administrative, and meteorological issues. The local legislation was taken as a reference for the water quality standards of the effluent. The consequent design of the wastewater treatment system of  which 12 h HDT for the septic tank and 5 d for the CW were considered. The results appeared to be adequate in respecting the standards discussed before. Indeed, the system is expected to reach a COD and TSS removal more than 80% and 90%, respectively.

Since few studies related to the treatment of wastewater originating from small-scale centers for plastic sorting exist so far, some of the hypotheses were theory-based. If organizational issues currently impeding the CPS to be fully operational would be solved, further investigation will be planned to gather field-based results including wastewater quality analysis, water quality evaluation after each unit and the related impact, and monitoring  and analysis of the sludge accumulation in the septic tank. So far, the study represents an attempt to fill the existing gap in the literature and provides  support to practitioners in the field.

## Supplementary Information

Below is the link to the electronic supplementary material.

Supplementary file1 (DOCX 2877 KB)

## Data Availability

Not applicable.
