# Supplementary material for: Appropriate solid waste management system in Quelimane (Mozambique): study and design of a small-scale center for plastic sorting with wastewater treatment
Source: Waste Dispos Sustain Energy. 2022 Feb 21;4(1):49–62. doi: 10.1007/s42768-022-00091-6 (PMC8859929; doi:10.1007/s42768-022-00091-6)
Supplement: Supplementary file 1 — Supplementary file1 (DOCX 2877 KB) [file 42768_2022_91_MOESM1_ESM.docx]

Appropriate Solid Waste Management system in Quelimane (Mozambique): study and design of a small-scale center for plastic sorting with wastewater treatment

Francesca Villa ^1,^*, Giovanni Vinti ^2^ and Mentore Vaccari ^2^

^1^ Department of Civil and Environmental Engeneering (DICA), Politecnico di Milano, via Golgi 39, Milan 20133, Italy

^2^ Laboratory Centre on Appropriate Technologies for Environmental Management in Resource-limited Countries (CeTAmb LAB), University of Brescia, via Branze, Brescia, Italy

* Corresponding author: Francesca Villa Tel: +39 3490982525, Fax: +, E-mail: [francesca.villa@polimi.it](mailto:francesca.villa@polimi.it), https://orcid.org/0000-0001-8630-3288

Supplementary materials – Annex n.1

This Annex contains the pictures of the center for plastic sorting (CPS) and the related wastewater treatment plant (WWTP).

**Fig. 1:** Layout of the center for plastic sorting.

| 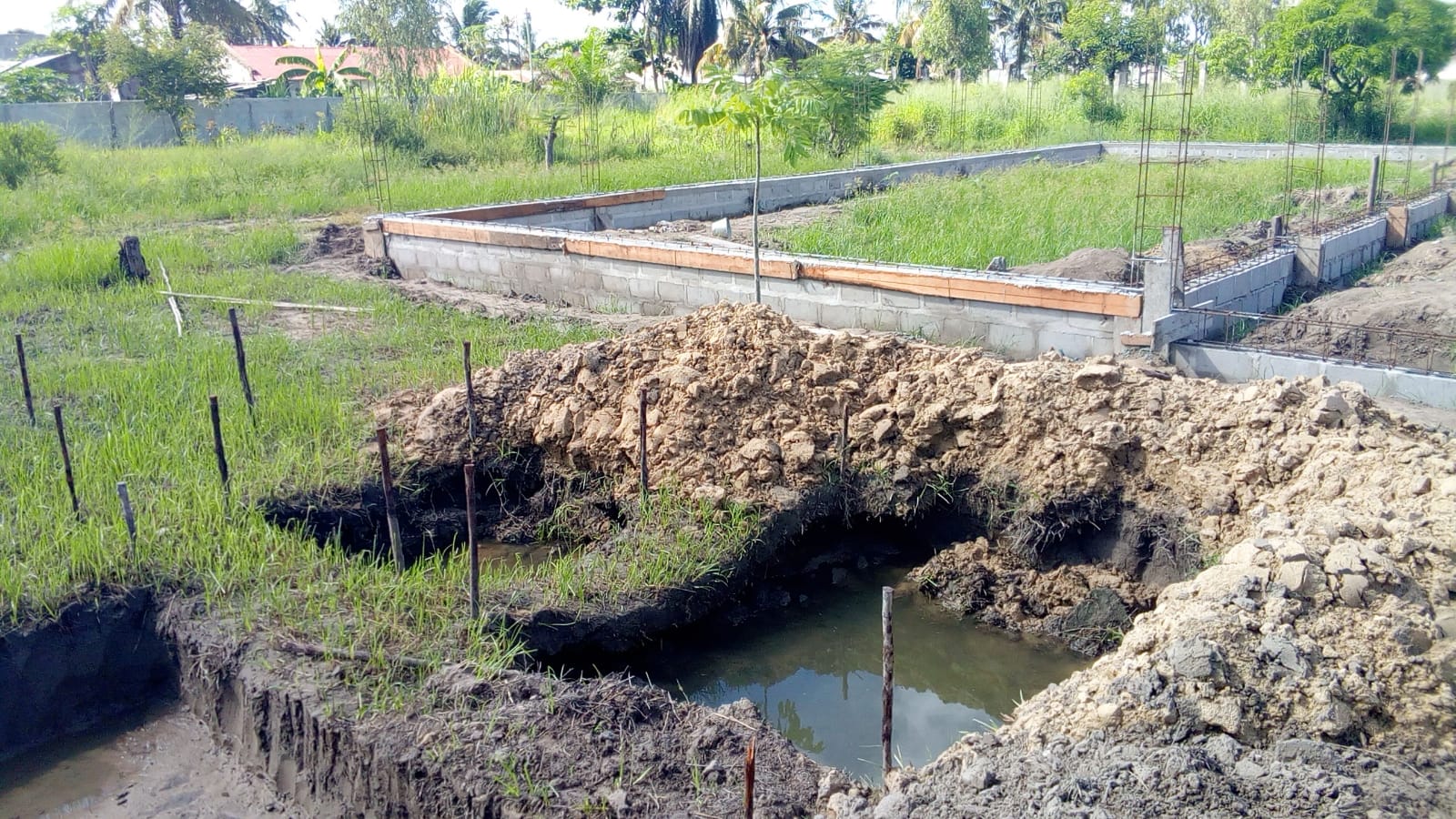 | **Fig. 2:**  Excavation works. Front line: septic tank and grease trap. Background: the main building of the CPS. (February 2019) |
| --- | --- |
| 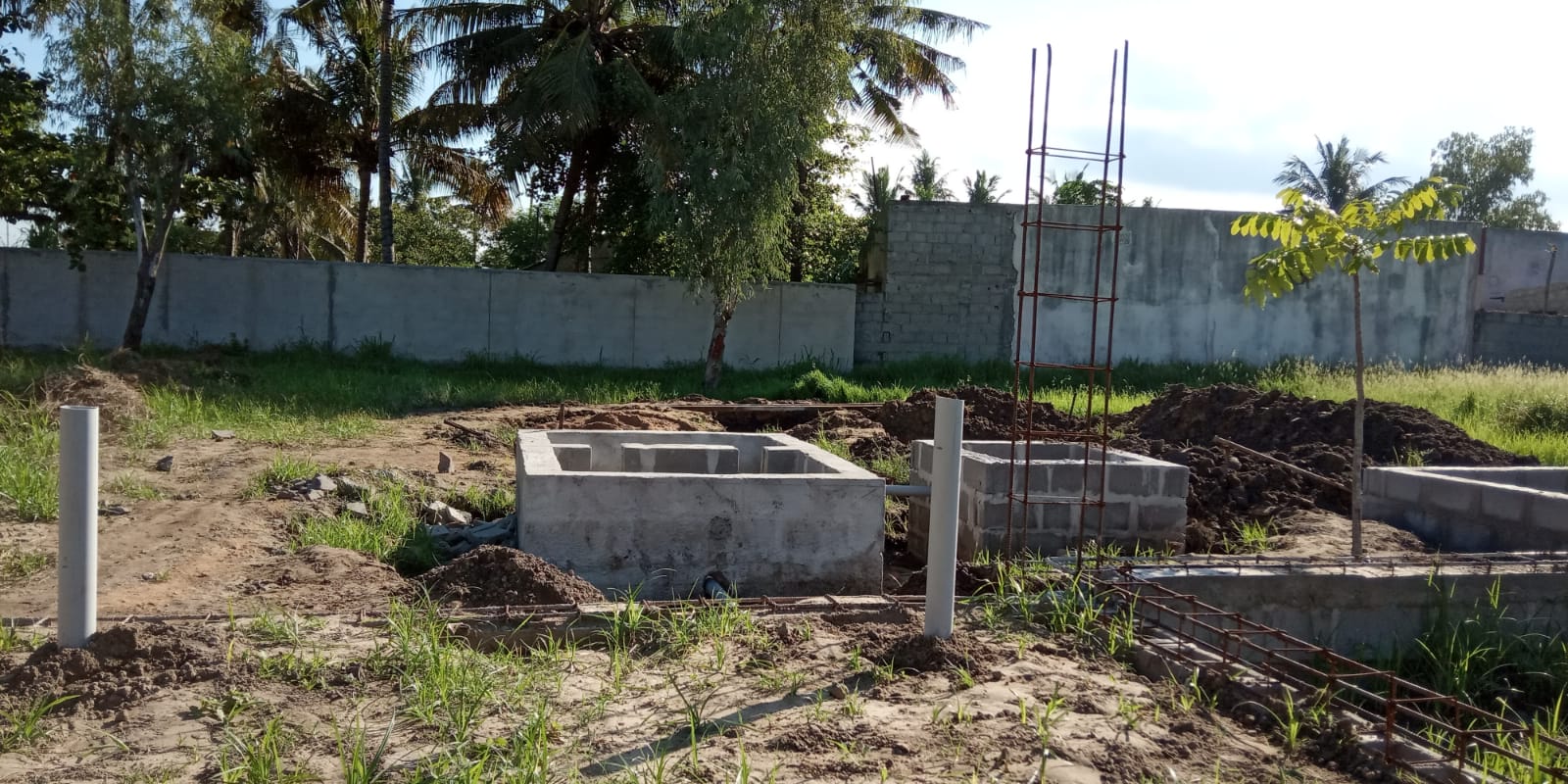 | **Fig. 3:**  Concrete works: septic tank and grease trap. (February 2019) |
| 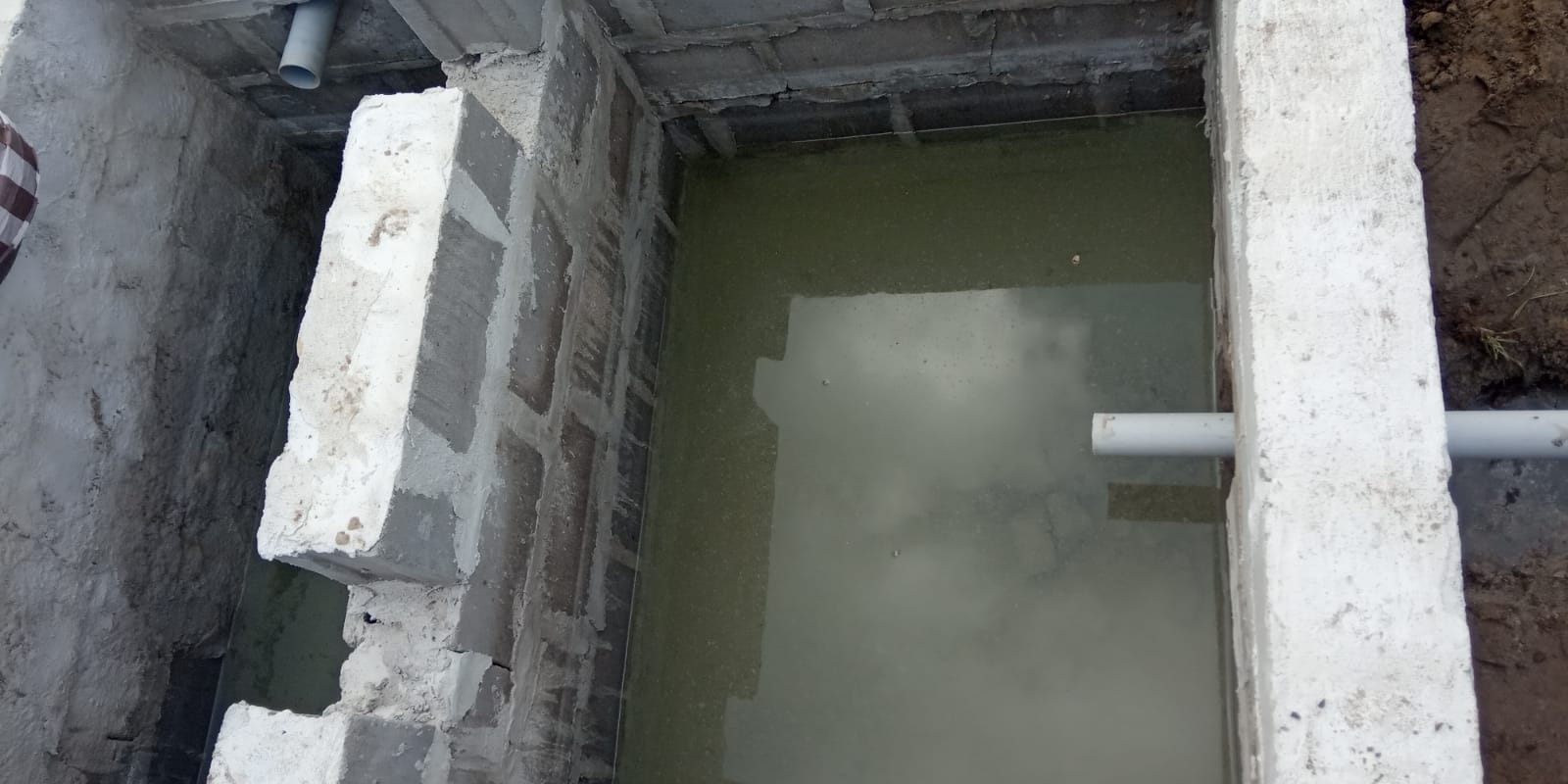 | **Fig. 4:**  Septic tank, right to left: connection coming from the washing tanks, first batch, sept with two openings, second batch, connection to the grease trap. (February 2019) |
| 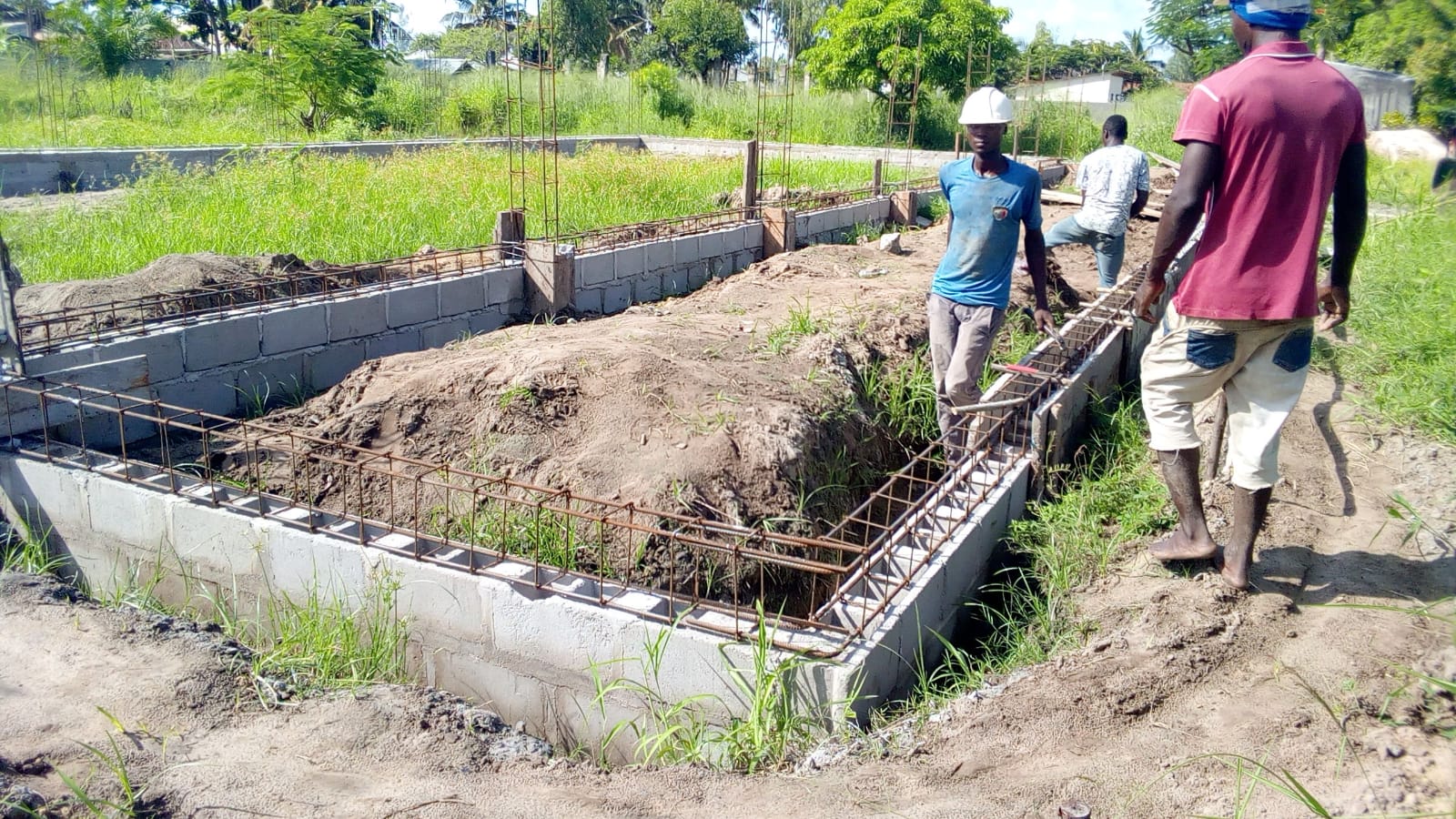 | **Fig. 5:**  Civil works. The main building of the CPS (left) and the drying area (right). (February 2019) |
| 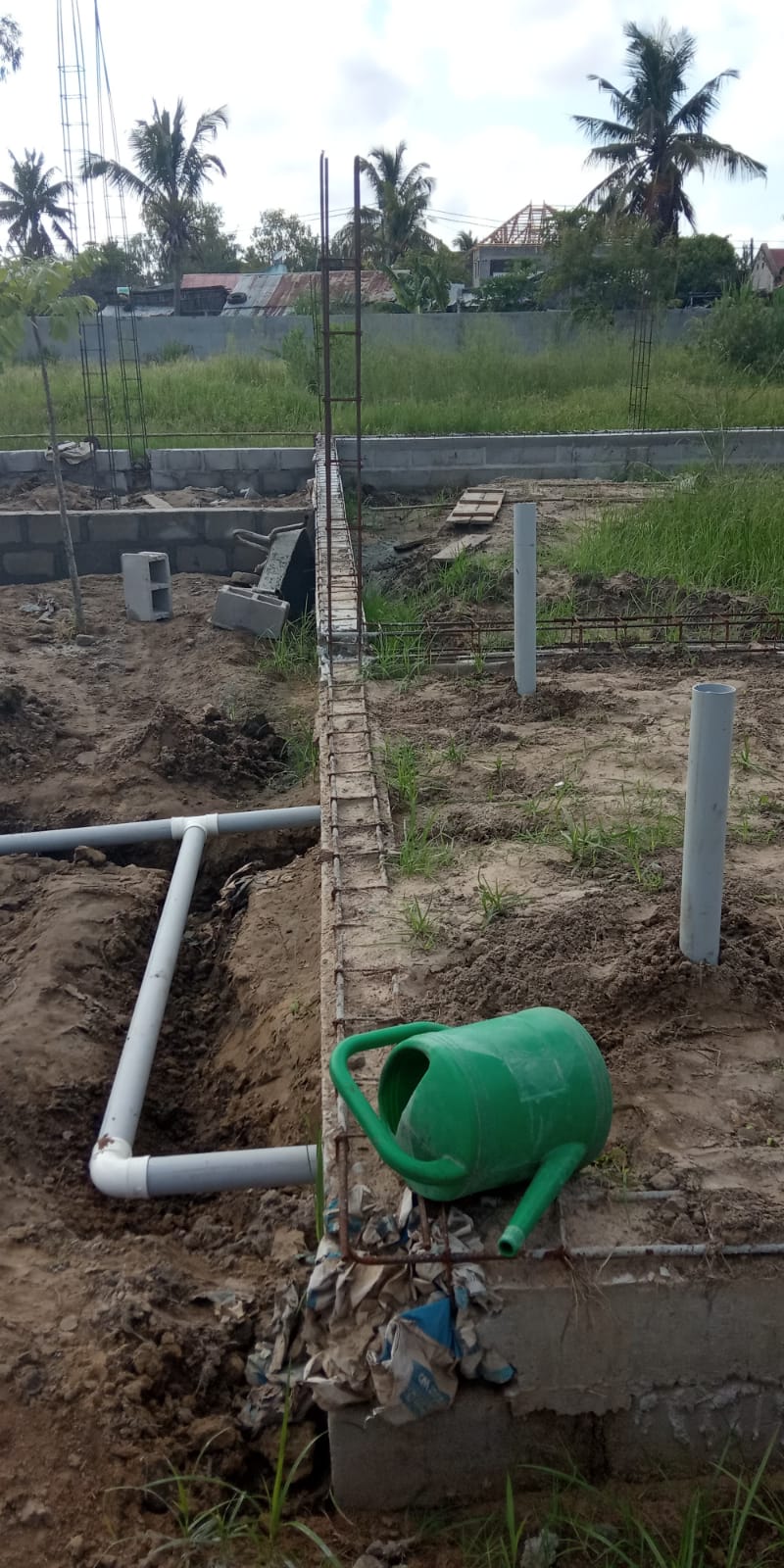 | **Fig. 6:**  Wastewater treatment plant, right to left: sewers of the washing tanks, pipes reaching the septic tank. (February 2019) |
| 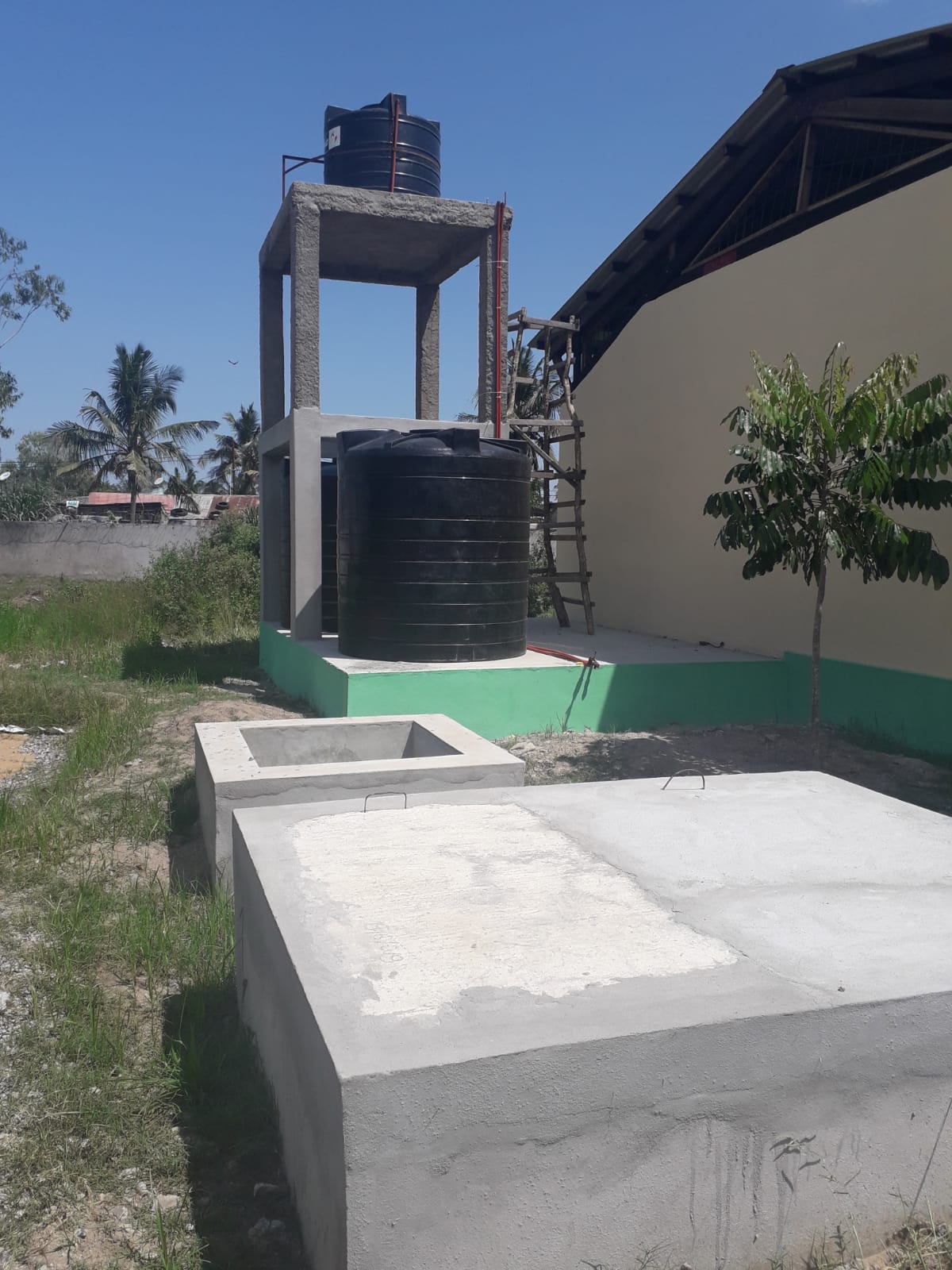 | **Fig. 7:**  Septic tanks and grease traps (completed), water tanks and main building of the center for plastic sorting. (June 2019) |
| 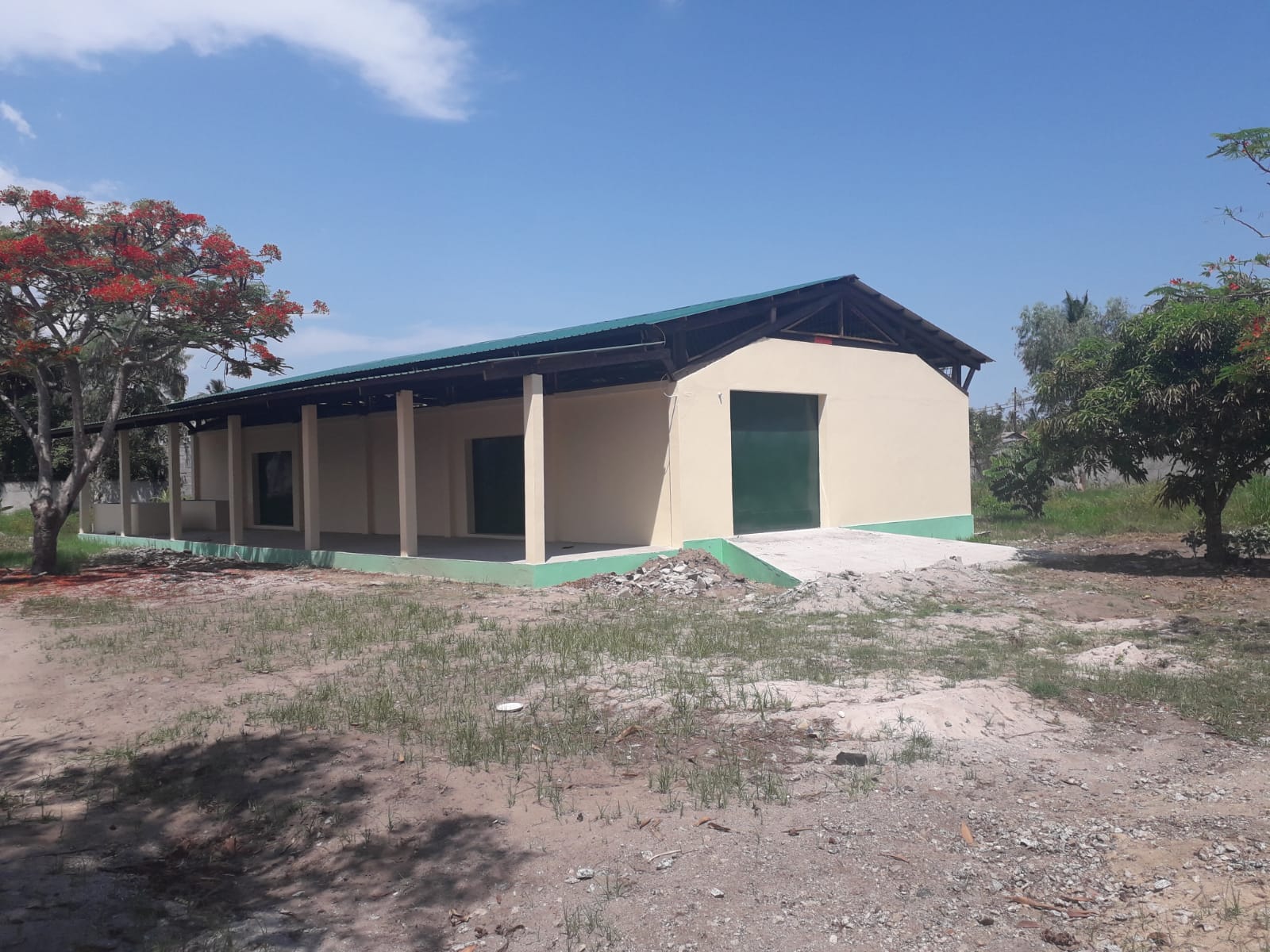 | **Fig. 8:**  The main building and the drying area. (June 2019) |
| 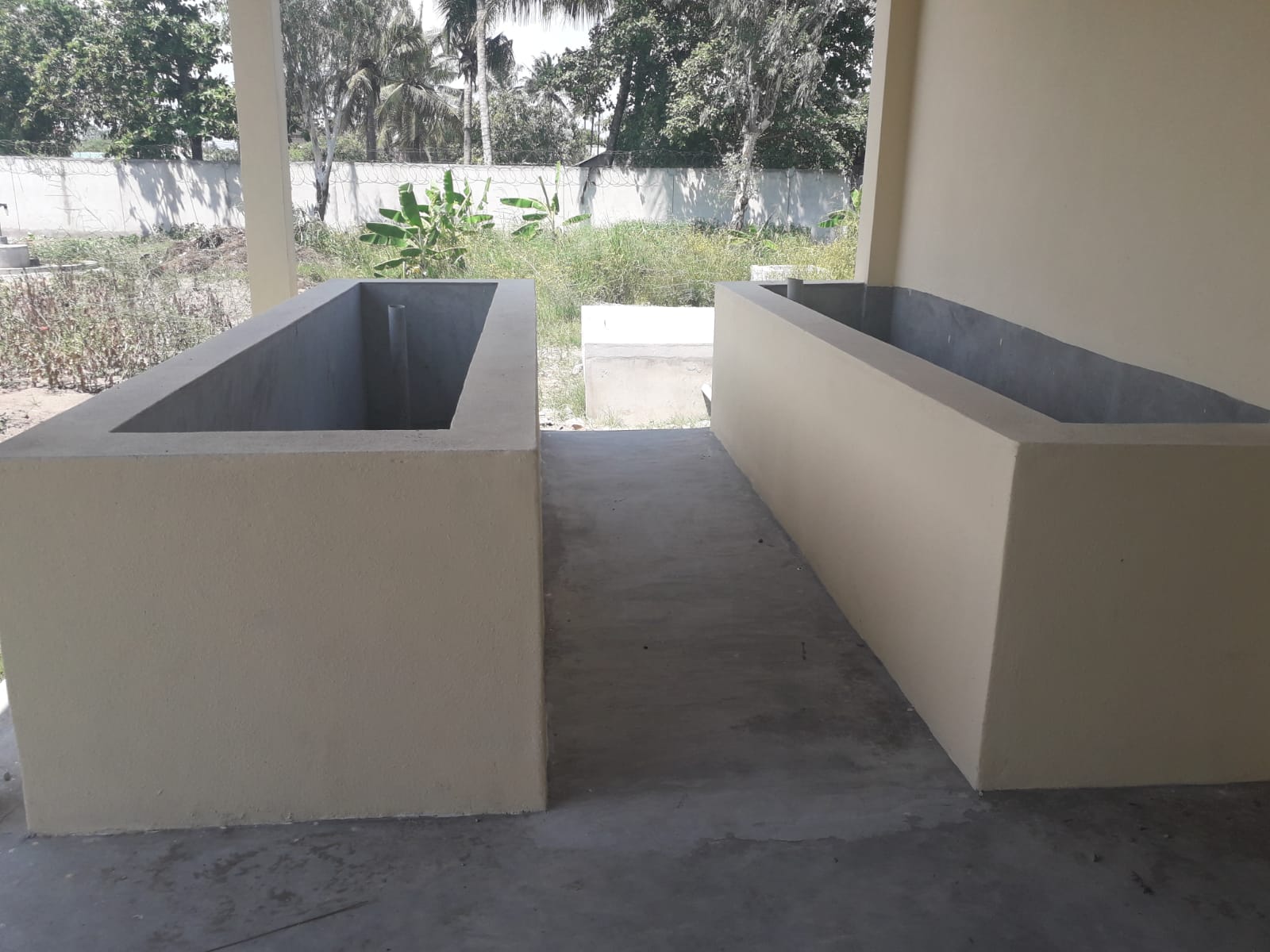 | **Fig. 9:**  The two washing tanks. (June 2019) |
| 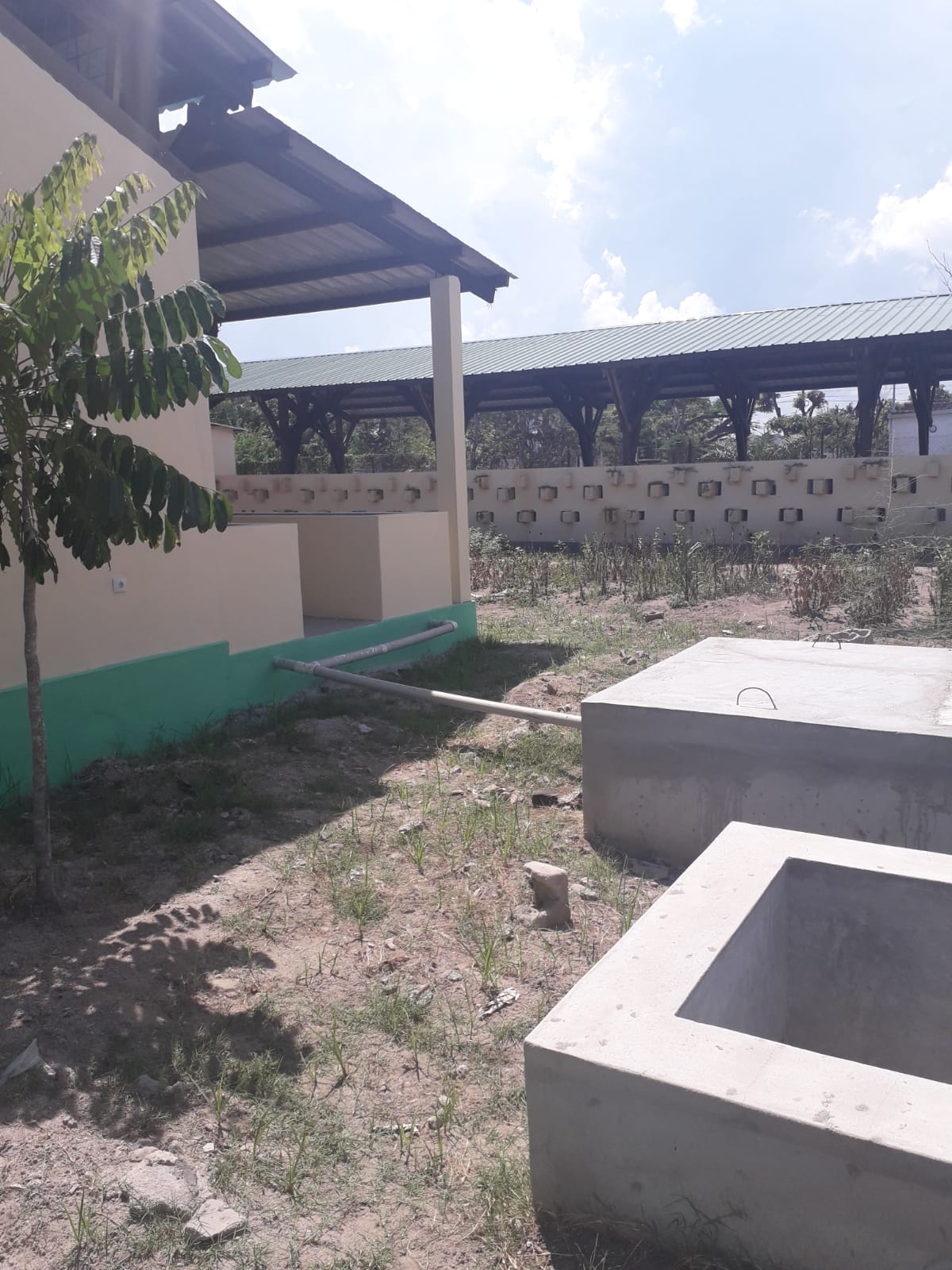 | **Fig. 10:**  Connection between the washing tanks located in the washing / drying area, and the septic tank. (June 2019) |
| 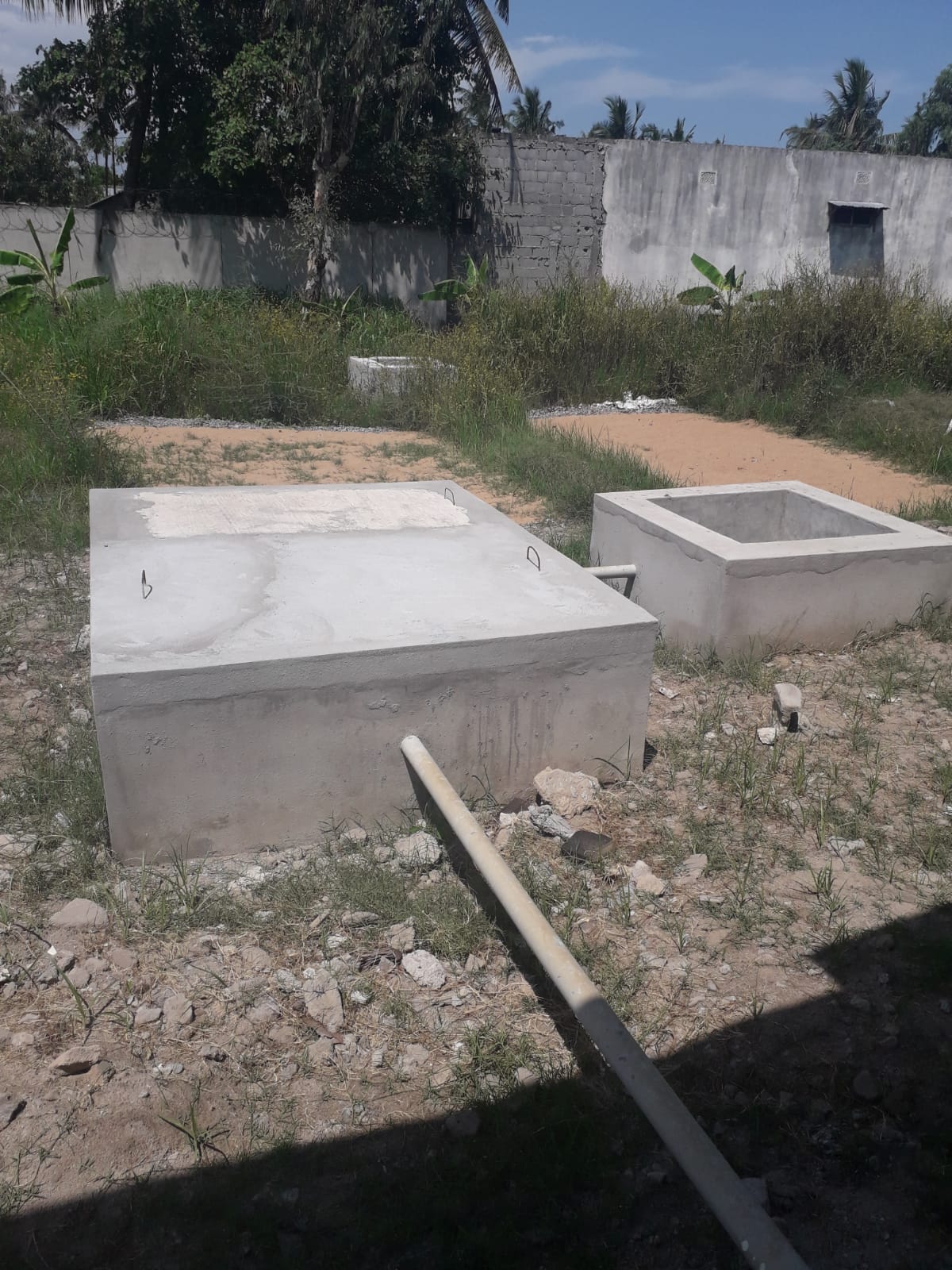 | **Fig. 11:**  Overview of the wastewater treatment plant (WWTP). Front: septic tank (left), grease trap (right). Back: SSF constructed wetlands. (June 2019) |
